# Building Appreciation for Natural History Collections from Biological Collection Data

**DOI:** 10.1093/iob/obag063

**Published:** 2026-10-21

**Authors:** G J Martin, J L Jensen, A Phillips, C McDonald, B Cunningham, S M Bybee

**Affiliations:** Department of Biology, 4102 LSB, Brigham Young University, Provo, UT 84602, USA; Monte L. Bean Life Science Museum, Brigham Young University, Provo, UT 84602, USA; Department of Biology, 4102 LSB, Brigham Young University, Provo, UT 84602, USA; Department of Preparatory Learning, School of Medicine, Brigham Young University, Provo, UT 84602, USA; Department of Biology, Utah Valley University, Orem, UT 84058, USA; Department of Biology, 4102 LSB, Brigham Young University, Provo, UT 84602, USA; Department of Biology, 4102 LSB, Brigham Young University, Provo, UT 84602, USA; Department of Biology, 4102 LSB, Brigham Young University, Provo, UT 84602, USA; Monte L. Bean Life Science Museum, Brigham Young University, Provo, UT 84602, USA

## Abstract

Recent calls have urged educators to involve students in the process of science to better engage them in learning. In this study, we investigate the effectiveness of using natural history collections (NHCs), a resource with which most students are unfamiliar, as tools for enhancing undergraduate teaching and shaping student attitudes toward both scientific resources and environmental issues. An inquiry-based module centered on firefly specimen data was implemented in introductory non-majors biology courses. The module aimed to (1) increase students’ perceived value of NHCs, and (2) improve pro-environmental attitudes as measured by the Revised New Ecological Paradigm (R-NEP) scale, and (3) increase student concern regarding firefly conservation. Students engaged with real specimen data to explore historical and current firefly distributions in Utah and evaluate the impacts of human activity, such as light pollution. Pre- and post-assessments revealed significant increases in student awareness of and attitudes toward the value of NHCs. However, improvements in environmental attitudes were limited, with modest gains in R-NEP items related to ecological concern. Additionally, students ranked fireflies lower in conservation priority compared to more charismatic or ecologically familiar organisms such as pandas and moss. These findings suggest that while NHC-based modules were associated with increased reported appreciation for collections as scientific resources, they may require more refinement to produce more meaningful changes in environmental attitudes. Importantly, the study highlights the pedagogical potential for NHC data for active engagement in the classroom.

## Introduction

There is a growing and documented need for inquiry-driven, active science education (National Research Council 2003; Brewer and Smith 2011; Cook et al. 2014). Engaging students in the practices of science, such as asking investigable questions, seeking answers through evaluating empirical data, and drawing evidence-based conclusions (Pedrosa-de-Jesus et al. 2012), a process ideally beginning in their K-12 science learning (Bybee 2011), allows students to begin to identify as scientists (Carlone and Johnson 2007; Jiang and Wei 2023). One of the available routes to engagement with science is through natural history collections (NHCs). These collections act as a historical repository showing how various organisms respond to environmental changes (Suarez and Tsutsui 2004; Winker 2004; Wandeler et al. 2007). In addition to examining the impact of environmental changes, NHCs can also be harnessed to conduct genetic studies (e.g., Neanderthal genomics; Prufer et al. 2014) to understand our own human history in addition to the history of the organisms with which we share the planet. Worldwide, NHCs house approximately 2–4 billion specimens (Arino 2010).

Natural history museums hold widely valued public-facing exhibits and activities. However, in addition to these publicly accessible displays, the non-public side of natural history museums include collections and laboratories that are not as accessible or fully understood by the general public (Suarez and Tsutsui 2004). This network of non-public collections represents a vast and largely untapped resource for the improvement of biology education. These collections offer a wealth of empirically investigable data in the form of specimens with which students can carry out scientific investigations, going beyond simply mirroring the process of science (Handelsman et al. 2004) toward actively asking and investigating scientific questions. NHCs provide an opportunity to increase students’ engagement in inquiry-driven science while also building a more complete understanding of the existence and value of collections among students that represent a subset of the general public.

The purpose of this study was to engage undergraduate students in an investigation relying on specimen data housed in a university NHC where the study took place. This module focused on two objectives, both aimed at undergraduate students in non-major introductory biology courses. The first of these was to increase student perception of the importance of NHCs. Our second objective was to increase pro-environmental thinking as assessed by the Revised-New Environmental Paradigm scale (R-NEP; Dunlap and Van Liere 1978, Dunlap et al. 2000; Valle et al. 2020). We chose to engage students using a module focused on human impacts on fireflies, to address the following questions:

1. How does engaging in a module using NHC data change undergraduate students’ value for NHCs?
2. How does engaging in a module using NHC data change undergraduate students’ environmental attitudes?
3. How does engaging in a module using NHC data, specifically with fireflies, change student concern regarding the endangerment and conservation of fireflies?

## Literature review

This section provides necessary background on specimens in science, specifically those found in NHCs. This is followed by a discussion of how scientific specimen data can be used for educational purposes. Finally, prior research on the development and use of modules using NHC data is discussed.

### Specimens in science

Specimens in NHCs are critical to scientific research by providing a repository of biodiversity and associated ecological data, as well as time stamps that allow scientists to track changes among organisms, dynamically, over time (Elith et al. 2006). For example, measuring population change and historical biogeography is usually difficult given a lack of historical data to compare with current populations. This problem is alleviated via the use of NHCs to reconstruct aspects of a species’ natural history, such as historical biodiversity (Shirey 2018; Tarli et al. 2018; Meineke et al. 2019; Whitaker and Kimmig 2020; Erickson and Smith 2021; Meineke and Daru 2021). For many taxa, these collections contain specimens dating to tens and hundreds of years ago (Shaffer et al. 1998; Shaw et al. 2004; Vik et al. 2008; Jeppsson et al. 2010). NHCs are also a resource for identifying and investigating invasive species and methods for controlling them (Suarez and Tsutsui 2004; Lister 2011). Historical data contained in NHCs about invasive species allows for the potential to locate biological control agents (e.g., predators, viruses, etc.) providing a critical evolutionary and historical perspective (Crawford and Hoagl and 2009). Specimens from NHCs are perhaps best known for their use in the discovery of new species; in a revision of the Australian beetle *Deretaphrus*, Lord and McHugh (2013) examined 1900 specimens across 44 museums and discovered seven previously undescribed beetles. In a similar study of Australian tardigrades, 11 new species were described (Claxton 1998). This result is not unusual, as most new species have been identified from specimens deposited in NHCs (Green 1998; Fontaine et al. 2012). From 2023 to 2024, at least 190 new species were described from one NHC alone, including a dinosaur (Lockwood et al. 2024), two piranhas (Machado et al. 2024), a snake (Mirza et al. 2024), and a moth (Sterling et al. 2024).

The longitudinal nature of NHCs (e.g., series of specimens collected over extended periods of time) allows for further comparative studies testing hypotheses focused on topics like climate change and/or habitat loss. In a study of passerine birds in Israel, Yom-Tov (2001) used NHCs to measure several species for both body mass and tarsus length and found a trend of decreasing body size from the years 1950 to 1999, coinciding with an increase in average summer temperature of 0.26°C. Climate change is known to affect many physiological aspects of an organism (Hughes 2000) and represents a potential explanation for this trend. Similarly, in a study of stream-inhabiting beetles, it was shown that an increase in size, coupled with a change to a more streamlined body, had occurred during a 60-year period and that a decrease in mean annual temperature was the most predictive variable for this trend (Babin-Fenske et al. 2008). Such studies would be infeasible without NHCs.

### Specimens in science teaching

In addition to the potential for discovery in specimen collections for practicing scientists, specimen-based learning has potential as an educative tool in formal undergraduate education. Specimens bring concrete, authentic life into the classroom, helping students appreciate that the concepts they are learning apply to living organisms. Engaging students in authentic science through the use of specimens alleviates the misconception that biology learning consists entirely of rote memorization (Handelsman et al. 2007). Using specimens and specimen metadata can drive inquiry-based investigation (Cook et al. 2014), allowing students to engage in scientific practices. The use of specimen data from NHCs can also potentially increase student understanding of what NHCs are and why they are important to preserve.

Traditionally, exposure to biological specimens in formal university biology education settings is usually limited to upper-level “-ology” type courses (e.g., entomology, botany, ornithology). Students in these classrooms learn first-hand the beauty and excitement hidden in the natural world (Wallace 1869). Despite these benefits, these experiences are rarely included in introductory-level biology courses (Powers et al. 2014). Even upper-level “-ology” courses, while more likely to use specimens, rarely focus on the importance of the NHCs from which they came and the scientific data that can be collected from them.

NHCs provide an opportunity to use specimens as a means of increasing awareness of diversity as they provide a way to interact with biodiversity on a personal level (Efthim 2006; Pickering et al. 2012; Kimble 2014; Monfils et al. 2017; Mabry et al. 2024, 2025). Insects are one of the most diverse groups of life and thus offer a choice opportunity to teach students about biodiversity (Wilson 1992; Mora et al. 2011). When most students think of insects, they may only think of the most common varieties that they regularly see or interact with, for example, butterflies, ants, bees, cockroaches, and mosquitos. With more than one million described species, and perhaps as many as 30 million species total (Erwin 1982), insects offer an opportunity for students to engage in inquiry about that which they are unfamiliar but which has large potential for discovery. Insects make up the majority of the holdings in many of the world’s NHCs (Holmes et al. 2016). In addition to the physical specimens, perhaps more important/useful in the classroom is the data associated with each specimen, such as the label. Label data ideally contains general locality information, and for more recently collected specimens, specific GPS coordinates. Most modern labels will and should also reference the date of collection, the collector, as well as any special collecting techniques or host plant information, although we note that some labels are incomplete. We included only complete label data. This wealth of information allows researchers to investigate questions with great specificity and provides students with the same opportunity.

The specimen metadata from NHCs is of great use to educators. In many instances, the use of actual specimens is too difficult; travel to a collection can take too long, and the loan of necessary specimens can exceed the ability of local collection managers. In these cases, the metadata can be used, as it is digitally available in several databases, housed across the world. There are thousands of data points that instructors can use as a context to bring scientific processes into the classroom. See, for example, the databases iDigBio (https://www.idigbio.org/) and GBIF (https://www.gbif.org). Through these databases, for example, anyone can download datasets containing the records of hundreds of firefly sightings recorded in many major NHCs across the United States. Included in these data are observations of locality, date, time of day, and in many cases, other useful information such as type of vegetation and/or habitat. This information can be curated and brought into the undergraduate classroom to investigate patterns of firefly distribution. Students can be challenged to develop hypotheses that would explain the data and to draw predictions from these hypotheses. Other, more locally focused, independent databases (e.g., https://nhmu.utah.edu/fireflies, based largely on museum specimens) can then be consulted. Using such resources, students can engage in scientific processes, forming hypotheses and analyzing data, in an authentic and personally relevant context.

### Developing modules to utilize NHCs

One instructional strategy for utilizing the wealth of data in NHCs is the development of modules for educational use. Modules are especially useful for engaging students in place-based and culturally relevant learning connected to nature, as has been shown through studies of native students’ learning from designed modules (e.g., Castagno and Brayboy 2008; Kisker et al. 2012; Barnhardt 2014; Lipka et al. 2014; Biological Collections Ecology and Evolution Network [BCEENET, n.d.]). In order to employ “big data” to engage undergraduates in testing authentic hypotheses, Lacey and colleagues (2017) created a module focused on the impacts of climate change on chipmunk populations using data found in NHCs sourced from Yosemite National Park. Similarly, Anderson et al. (2017) created a museum-based module employing place-based learning using collections data from the University of Alaska Museum. These studies illustrate the utility of NHCs for creating relevant learning experiences for students and non-students alike. Following from what has been shown, this study also employs NHC data to develop a module focused on the local, relevant, and place-based phenomenon of firefly habitat loss.

## Methods

### Participants

Participants for this study were undergraduates selected from introductory non-majors biology courses (*n* = 267) at a large, private institution in the Western United States. This course consists of freshmen to seniors and is part of the General Education requirement at the University. All participants gave their consent to participate in the study, which received approval from the Institutional Review Board (# E19292). The module was given as a required class activity with points attached to participation.

### Module

The online module was designed with firefly specimen data from the Monte L. Bean Life Science Museum (MLBM) at Brigham Young University. Fireflies were chosen as the module organism because of their status as a charismatic and well-known species (Albert et al. 2018), their gender-neutrality (i.e., they are equally liked or disliked by both male and female students; Mota Pereira et al. 2023), and because they are of rare occurrence in Utah and at risk of extirpation. Fireflies are also an area of expertise for two of the researchers conducting this study.

The primary goals of the module were threefold. The first goal was to increase student understanding of and value for NHCs as a resource. The second goal was to increase students’ environmental thinking and attitudes generally. Finally, the third goal was to increase student concern regarding the endangerment and conservation of fireflies.

To address the first goal, the module began with a brief description of the MLBM and the primary purpose of its NHCs (i.e., to advance scientific knowledge, address societal issues, and to increase scientific literacy). Students were instructed about the major holdings of the MLBM and taught that one of the major strengths of the MLBM is the insect collection with over 2 million specimens. Then, to extend this idea toward the second goal of the module, students were given a brief background on fireflies. They learned that fireflies are beetles, many species rely on bioluminescence (use of light for communication) for sexual reproduction, and that because of this, fireflies are highly susceptible to light pollution.

To give students experience learning about fireflies using NHC data, students were given an Excel spreadsheet of curated data from MLBM specimens. They were asked to examine the data and determine where in Utah, and at what time of year, fireflies have historically been observed. Students were then given instructions on how to use the website simplemappr.net to obtain this information themselves. Finally, students were given a short assessment to determine whether they could correctly interpret the data. This assessment consisted of one multiple choice question: “If you wanted to go and see fireflies in Utah, where and when would you go?” Possible responses were: A. Lake Bottom Canal, Provo; last week of June, B. Goshen Marshes; first week of June, C. Spring Lake, Payson; last week of February, and D. Dugway; first week of July. The data given to the students represented all locations except Dugway, and all times of year except February. This was designed to lead students to select options A or B.

If students chose option A, then they were congratulated on selecting a correct answer. However, this was followed with the information that while fireflies are historically known at this location from independent community reports and expert observation, Lake Bottom Canal has not had fireflies for several years, if not decades. Students were presented with images of what the location looks like present-day, bordered by a freeway with large lighted billboards and streetlights. Students were asked to hypothesize why fireflies are no longer found at this locality. They were then directed to the slides for option B. If students originally chose option B, then they were congratulated for choosing a correct answer and presented with a short video clip of fireflies in Utah. They were then directed to the slides for option A. The redirection ensured that students would be able to compare two different locations and whether they currently provide suitable firefly habitat. After seeing both options, students were asked to compare the two habitats. Then, moving toward the third goal of the module regarding students’ environmental attitudes, students were asked to think about the unintended consequences of human actions and infrastructure on nature, such as the consequences of light pollution. This was followed by a series of thought questions and statements illustrating the role of NHCs on our understanding of changing animal populations. To conclude the module, students were asked, “When human development is at odds with the natural world, what, if any, is our responsibility to protect it?” Students were scored for participation and completion of the module, rather than for the answers they chose and the order in which they chose them.

### Instruments

In this study, we sought to measure changes in students’ attitudes toward NHCs and their environmental attitudes in general. To measure the first goal, two Likert-scale items were included that asked for students’ agreement to the following statements: (1) NHCs are important for scientific research, and (2) NHCs are important for the general public. To measure students’ environmental attitudes, we administered the R-NEP (Dunlap et al. 2000) survey which measures pro-environmental attitudes using 15 Likert-scale items. All Likert-scale items were on a five-point scale ranging from Strongly Disagree to Strongly Agree. Even items were reverse coded. Because the module centered on fireflies in the Utah region, four questions were added to investigate changes in students’ attitudes toward fireflies compared to another well-known charismatic endangered species (i.e., the panda; Albert et al. 2018), and a plant species (i.e., moss). We asked students, “On a scale of 1 (no concern) to 10 (extreme concern) how concerned are you with the extinction of [fireflies, pandas, moss]?” This was followed by a question asking students, “In your opinion, how would you rank the above three organisms (fireflies, pandas, and moss) in the importance of preventing them from going extinct?” Students responded to these questions pre- and post-participation in the online module.

### Analysis

The 17 Likert-scale questions were compared pre- and post- using a paired-sample *t*-test implemented in SPSS (IBM; Version 28.0.0.0). Additionally, the 15 R-NEP scale questions were divided into the five facets of an ecological worldview (Dunlap et al. 2000): the reality of limits to growth, anti-anthropocentrism, the fragility of nature’s balance, rejection of exemptionalism, and the possibility of an ecocrisis. Totals were calculated by summing the averages for the three items in each facet. The total score was calculated by summing the averages to all 15 items.

For the items asking students to rank how concerned they were with an organism’s extinction, we calculated an average rank for each species, fireflies, pandas, and moss. For the item asking them to rank the importance of preventing extinction, we tallied how often each organism was ranked in the first priority spot.

## Results

The number of students who completed both the pre- and post-assessment was 267. Table 1 displays the *t*-test results for each of the R-NEP items, as well as the five R-NEP facets of an ecological worldview (Dunlap et al. 2000) and the total R-NEP score prior to and directly following the module.

**Table 1:** *T*-test results for pre- to post-intervention survey items

Survey item	Pre-test average	Post-test average	*P*-value	Cohen’s *d*
1. We are approaching the limit of the number of people the Earth can support.	2.69	2.79	0.083	−0.107
2. Humans have the right to modify the natural environment to suit their needs.	3.41	3.40	0.866	0.010
3. When humans interfere with nature it often produces disastrous consequences.	3.45	3.61	0.012*	−0.155
4. Human ingenuity will ensure that we do not make the Earth unlivable.	3.15	3.20	0.383	−0.054
5. Humans are seriously abusing the environment.	3.82	3.65	0.005**	0.176
6. The Earth has plenty of natural resources if we just learn how to develop them.	3.88	3.86	0.806	0.015
7. Plants and animals have as much right as humans to exist.	3.75	3.89	0.032*	−0.132
8. The balance of nature is strong enough to cope with the impacts of modern industrial nations.	2.52	2.59	0.224	−0.075
9. Despite our special abilities, humans are still subject to the laws of nature.	4.25	4.20	0.395	0.052
10. The so-called “ecological crisis” facing humankind has been greatly exaggerated.	2.82	2.85	0.582	−0.034
11. The Earth is like a spaceship with very limited room and resources.	2.86	2.96	0.101	−0.101
12. Humans were meant to rule over the rest of nature.	3.54	3.56	0.812	−0.015
13. The balance of nature is very delicate and easily upset.	3.26	3.34	0.154	−0.088
14. Humans will eventually learn enough about how nature works to be able to control it.	2.85	2.87	0.697	−0.024
15. If things continue on their present course, then we will soon experience a major ecological catastrophe.	3.41	3.40	0.839	0.012
16. Natural history collections are important for scientific research.	4.08	4.29	<0.001**	−0.235
17. Natural history collections are important for the general public.	3.90	4.14	<0.001**	−0.261
Construct totals: (Even items have been reverse-coded)^a^				
R-NEP ecological worldview: the reality of limits to growth (sum of items 1, 6, and 11)	7.67	7.88	0.078	−0.109
R-NEP ecological worldview: anti-anthropocentrism (sum of items 2, 7, and 12)	8.79	8.93	0.236	−0.073
R-NEP ecological worldview: the fragility of nature’s balance (sum of items 3, 8, and 13)	10.19	10.36	0.148	−0.090
R-NEP ecological worldview: rejection of exemptionalism (sum of items 4, 9, and 14)	10.25	10.14	0.286	0.066
R-NEP ecological worldview: possibility of an ecocrisis (sum of items 5, 10, and 15)	10.40	10.18	0.049*	0.123
Total (sum of items 1–15; range: 15–75)	47.11	47.32	0.494	−0.044

Note: Each item was on a 5-point Likert scale. Numbers represent average agreement with 1–2 indicating disagreement, 3 indicating a neutral stance, and 4–5 indicating agreement. Construct totals were calculated by adding the three item averages for each construct according to Dunlap et al. (2000); higher numbers would indicate greater agreement; range is 3–15. * indicates significance *P* < 0.05, ** indicates significance *P* < 0.01.

^a^With reverse-coding in construct totals, higher numbers indicate more “pro-environmental” viewpoints.

Analysis showed no significant differences in total score for four of the five facets of the R-NEP. The facet “possibility of an ecocrisis” (*P* = 0.049) was significantly lower (i.e., more agreement) post-module. Additionally, the total R-NEP score did not change from pre to post. When looking at individual items, there was a significant difference (*P* < 0.05) for the following items: “When humans interfere with nature it often produces disastrous consequences” (*P* = 0.012), “Humans are seriously abusing the environment” (*P* = 0.005), and “Plants and animals have just as much right as humans to exist” (*P* = 0.032). The highest change was in the items “Natural history collections are important for scientific research” (*P* < 0.001) and “Natural history collections are important for the general public” (*P* < −0.001; see Fig. 1). Note that this is a self-reported importance score and may not reflect a broader increase in intrinsic value to the students.

**Fig. 1:**
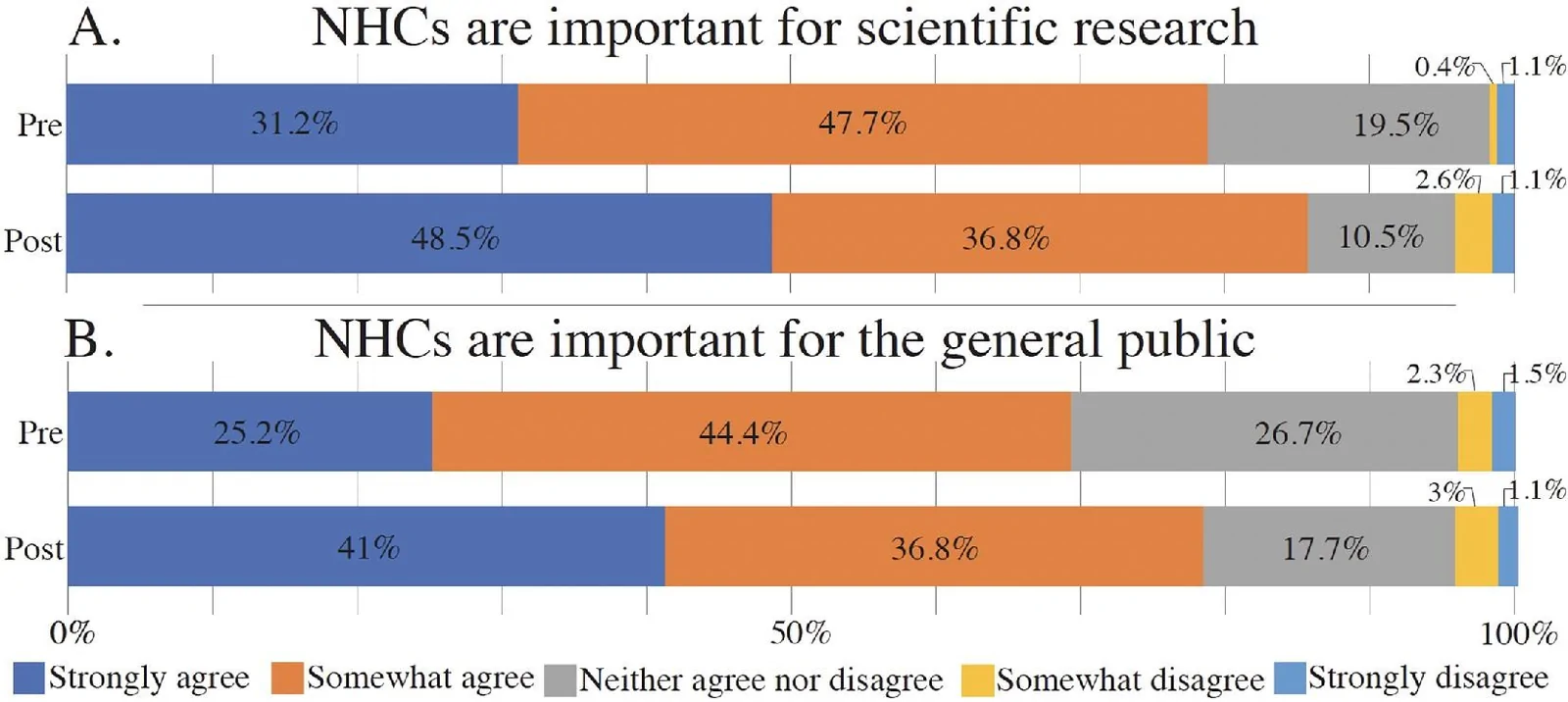
Distribution of responses for the survey item (A) “natural history collections are important for scientific research” and (B) “natural history collections are important for the general public” pre- and post-intervention (*N* = 265). Response categories are indicated in the figure legend. Percentages are provided in the bar.

After the intervention, when students were asked to rank, on a scale from 1 (no concern) to 10 (extreme concern), their concern over the extinction of fireflies versus a more charismatic species, the panda, and a plant species, moss, students ranked both pandas (average concern score of 6.56) and moss (average concern score of 5.91) higher than fireflies (average concern score of 5.59), with pandas being the highest ranked. However, when asked to rank order them in terms of the importance given to prevent their extinction, students most often ranked moss as top priority (52% of students), followed by pandas (32% of students), followed by “equally important” (9% of students), followed by fireflies (7% of students).

## Discussion

The first goal of the module intervention with undergraduate biology students was to increase their value for NHCs. Results indicated that students had significant increases in their agreement with the statements, “Natural history collections are important for scientific research” and “Natural history collections are important for the general public.” This significant increase took place despite the fact that students already had high agreement with these statements prior to the intervention (1.92 and 2.10, respectively, with numbers closer to 1 indicating higher agreement). Looking at actual responses, there was a notable shift in the composition of each response group. For example, for the pre-survey question addressing NHC importance for science, the majority of students responded either “somewhat agree” or “neither agree nor disagree.” Post-intervention, both of these groups diminished with a marked increase in the number of students responding “strongly agree” (Fig. 1). A similar pattern was observed for the question addressing NHC importance for the general public. Comparing the two questions, fewer students strongly agreed that NHCs were important for the general public (41% in the post-survey) compared to those who strongly agreed that NHCs were important for science (48% in the post-survey).

The second goal of the module intervention was to increase participants’ environmental attitudes. The gains seen in value for NHCs generally were not seen with regard to pro-environmental thinking as assessed by the R-NEP, that is, we saw no significant change in overall R-NEP scores. While individual NHC items showed statistical differences, only a few items related to environmental issues showed significant changes. There are several possible factors contributing to the lack of a shift toward pro-environmental attitudes. First, a large portion of the online module was dedicated to explaining NHCs. It is possible that this unbalanced attention resulted in failing to communicate overall risk to the environment. Given these findings, future research could examine the use of different examples of extirpation patterns in Utah to determine whether that could bring about a greater change in R-NEP scores. Second, it may be that one module in one class was not enough to influence attitudes toward the environment generally, especially among the student body in question. According to a recent poll, Brigham Young University is one of the only universities in the country where students are majority conservative (Stevens 2020). Additionally, the student body is almost entirely affiliated with the Church of Jesus Christ of Latter-day Saints whose membership is often associated with conservative ideologies. However, it should be noted that there is a diversity of viewpoints on the environment amongst Church membership, and recent writings from leaders would suggest a more pro-environmental viewpoint than many conservative religious populations in America (Handley 2016; Studer and Burge 2018; Brigham Young University 2025; Church of Jesus Christ of Latter-day Saints 2025).

The third goal of the module was to increase student concern regarding the endangerment and conservation of fireflies. Overall, the module did not improve the students’ perception of the importance of fireflies. Again, this may be due to the large emphasis given to NHCs and less specific emphasis on the importance of fireflies and firefly conservation. In fact, students reported the greatest level of concern for pandas, followed by moss, while fireflies elicited the lowest level of concern. When asked to prioritize species for conservation, students ranked moss as the highest priority, followed by pandas, and then “equally important,” fireflies were once again ranked lowest. Students were allowed to offer an explanation of their ranking in the free response section. Many students placed moss as the most important organism for conservation efforts because of their stated assumption that mosses are a more critical piece of the ecosystem. Similar to the emphasis placed on lichens as an indicator species (Thakur et al. 2024), perhaps students made the same connection with mosses. Or perhaps they are relating their understanding of the trophic food web to importance, with moss being seen as a primary producer and therefore at the base of the trophic structure and therefore most important. Additionally, fireflies are not widespread in Utah, and many students have likely never seen one. In fact, one student indicated their low ranking of fireflies by stating, “I’ve never seen one.” Additionally, nearly 60% of the student body comes from western states (Yfacts 2026), where fireflies are not as common as they are in the eastern and southern parts of the US (iNaturalist 2026). Thus, it may be just due to a lack of familiarity with fireflies that students did not rank them as high priority for conservation efforts.

The results indicate that charismatic species, such as pandas, are more likely to be favored over the less charismatic firefly and are more frequently perceived as higher priorities in conservation efforts (although moss were ranked highest in this study for reasons we have speculated about above). Visually appealing species that already benefit from greater public awareness and positive perceptions are associated with elevated levels of concern, particularly when compared to species that students have not previously recognized as relevant or important (Colléony et al. 2017).

This study aimed to show the value of using NHC data in the classroom as a means of increasing student appreciation for both the NHCs themselves and the environment more generally. A positive attitude toward NHCs is critical for the maintenance, expansion, and continued use, especially among future biologists who may utilize these collections in their own future research and conservation efforts as well as future citizens, educators, and policymakers who will influence conservation initiatives. This study illustrated the benefit of instructional materials utilizing NHC data to increase attitudes toward NHCs. More research is needed examining module use and NHC data to refine our ability to increase pro-environmental attitudes among students. NHCs provide a critical longitudinal resource for examining ecosystem health in the Anthropocene era, and increasing students’ pro-environmental attitudes by using these resources has great potential, and should be an area of future research.

## Data Availability

Data is available through BYU ScholarsArchive, https://scholarsarchive.byu.edu/data/115.
